# Coexistence of Atrioventricular Nodal Reentrant Tachycardia and Idiopathic Left Ventricular Outflow-Tract Tachycardia

**Published:** 2005-04-01

**Authors:** Majid Haghjoo, Arash Arya, Mohammadreza Dehghani, Zahra Emkanjoo, Amirfarjam Fazelifar, Alireza Heidari, MohammadAli Sadr-Ameli

**Affiliations:** Department of Pacemaker and Electrophysiology, Shahid Rajaie Cardiovascular Medical and Research Center, Iran University of Medical Sciences, Tehran, Iran

**Keywords:** double tachycardia, left ventricular outflow tract tachycardia, atrioventricular nodal reentrant tachycardia

## Abstract

Double tachycardia is a relatively rare condition. We describe a 21 year old woman with history of frequent palpitations. In one of these episodes, she had wide complex tachycardia with right bundle branch and inferior axis morphology. A typical atrioventricular nodal tachycardia was induced during electrophysiologic study, aimed at induction of clinically documented tachycardia. Initially no ventricular tachycardia was inducible. After successful ablation of slow pathway, a wide complex tachycardia was induced by programmed stimulation from right ventricular outflow tract. Mapping localized the focus of tachycardia in left ventricular outflow tract and successfully ablated via retrograde aortic approach. During 7 month's follow-up, she has been symptom free with no recurrence. This work describes successful ablation of rare combination of typical atrioventricular nodal tachycardia and left ventricular outflow tract tachycardia in the same patient during one session.

## Introduction

Double tachycardia, defined as the simultaneous occurrence of atrial and ventricular [1,2] or junctional and ventricular tachycardia (VT) [3], has been rarely reported and usually occurs in patients with poor left ventricular function or in association with digitalis intoxication [1,2,4,5]. The coexistence of atrioventricular reentrant tachycardia (AVRT) and idiopathic right ventricular outflow tract (RVOT) tachycardia [3], or atrioventricular nodal reentrant tachycardia (AVNRT) and RVOT-tachycardia [6] has also been reported, but coincidence of AVNRT and idiopathic left ventricular outflow tract (LVOT) tachycardia has rarely been reported. To the best of our knowledge, this case is the first report of successful ablation of rare combination of AVNRT and LVOT-tachycardia in the same patient.

## Case report

**Figure 1:**
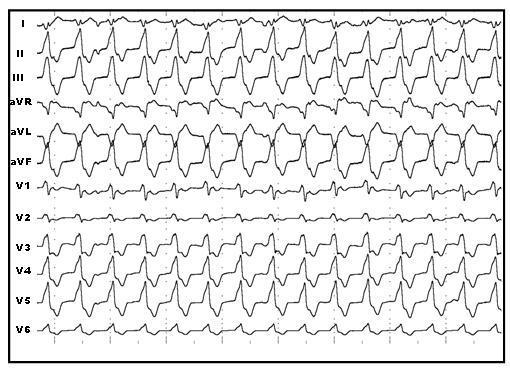
The standard 12-lead electrogram of wide QRS tachycardia (125 beats /min) showing right bundle branch block and inferior axis morphology compatible with left ventricular outflow tract tachycardia.

A 21 year old woman with no evidence of structural heart disease referred to our center for evaluation of palpitation and dizziness. The structural heart disease was excluded by physical examination and transthoracic echocardiography. Transthoracic echocardiography showed normal cardiac chambers (including right ventricle), normal valvular function and ejection fraction (EF) without any wall motion abnormalities. During an episode of palpitation, the standard 12-lead electrocardiogram (ECG) showed documented wide complex tachycardia with a heart rate of 125 beats /min. The tachycardia was refractory to two intravenous antiarrhythmics (amiodarone, procainamide). The wide complex tachycardia had inferior axis and right bundle branch block morphology compatible with LVOT-tachycardia (Figure 1). The baseline ECG showed no abnormality.

**Figure 2:**
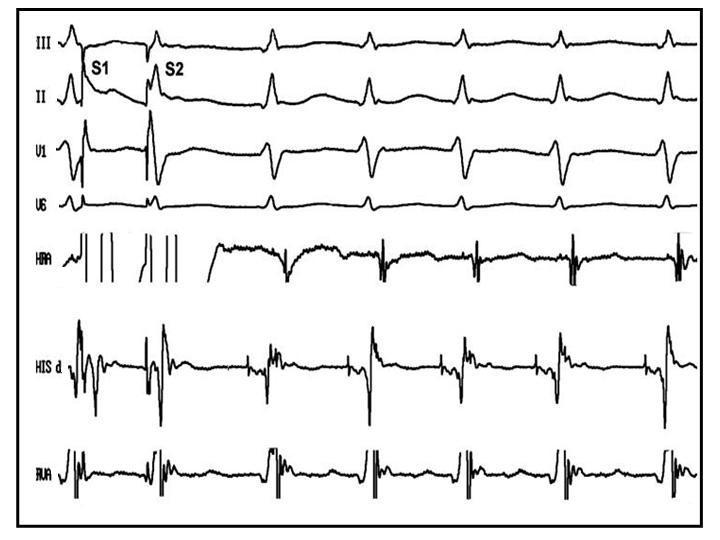
Electrophysiologic tracing of narrow complex tachycardia compatible with typical atrioventricular nodal reentrant tachycardia.

**Figure 3:**
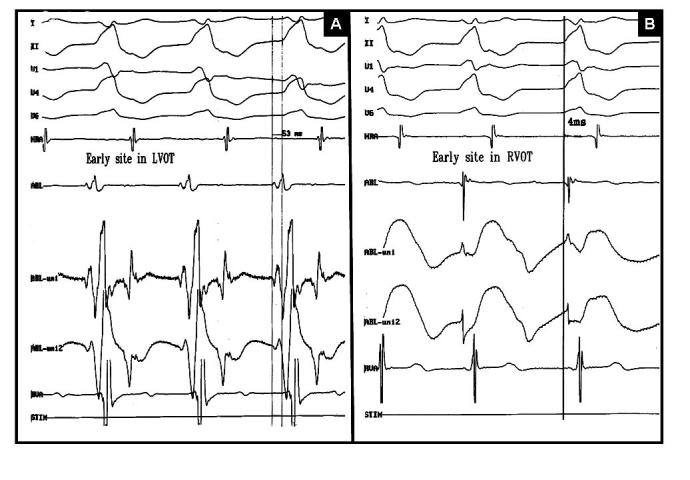
Demonstration of earliest activation site in left ventricular outflow tract (A) and right ventricular outflow tract (B) recorded during mapping by ablation catheter

**Figure 4:**
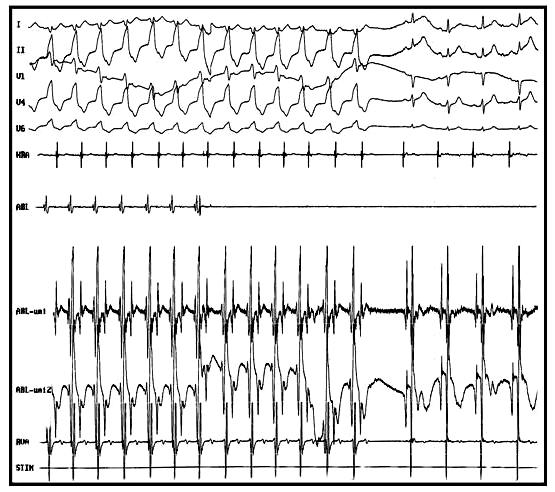
Termination of ventricular tachycardia by delivery of radiofrequency energy in early site in left ventricular outflow tract

After obtaining written informed consent, electrophysiologic study was done in the postabsorptive and nonsedated state. During programmed electrical stimulation from atrium and ventricle, dual AV nodal physiology with nonsustained AVNRT was induced. Then programmed ventricular stimulation was performed with standard protocol at three cycle length (600,500,400ms) and three extrastimuli up to coupling interval of 200 ms from two sites (RV apex, RVOT). No ventricular tachycardia was induced with and without isoproterenol infusion. Repeat programmed atrial stimulation resulted in induction of sustained AVNRT under isoproterenol infusion (Figure 2). Radiofrequency catheter ablation of slow pathway was done at right posteroseptal area. Postablation programmed stimulation failed to induce any supraventricular tachycardia with and without isoproterenol infusion but a wide complex tachycardia (cycle length=480 ms) identical to clinically documented arrhythmia was induced by overdrive pacing from RVOT. Mapping of RVOT failed to show any early ventricular activation site, thus LVOT was mapped and tachycardia focus was localized in this area with 53 ms early ventricular activation relative to surface electrocardiogram (Figure 3) Radiofrequency energy delivery (50 W, 60°C) at this site resulted in termination of tachycardia (Figure 4). Thirty minutes after ablation, no tachycardia was induced with and without isoproterenol infusion. During 12 month follow-up, she has been symptom free with no antiarrhythmic drugs.

## Discussion

Double tachycardia was a relatively uncommon type of tachycardia in previous reports [1,3,5]. In recent study by Kautzner et al [7], this combination (RVOT-tachycardia and AVNRT) was not as uncommon (15% of RVOT-tachycardia patients had AVNRT). In this study, three of seven patients with coexistent idiopathic ventricular outflow tachycardia and AVNRT had the arrhythmogenic focus localized in the uppermost part of the septum or more epicardially near the great cardiac vein as documented by detailed mapping but no early site was reported in LVOT area or aortic cusps in any patients. In this study, no attempt for ablation was made in the patients with epicardial variant.

Idiopathic VT most commonly arises from RV than LV (70% versus 30%) [8]. Idiopathic LVOT-tachycardia is one of the three subtypes of idiopathic left ventricular tachycardia that analogous to adenosine sensitive RVOT-tachycardia originate from deep within the septum and exit from left side of septum, and result from cAMP-mediated triggered activity [9]. AVNRT is a typical reentrant tachycardia originating from the AV nodal and perinodal tissues [10].

In this patient, clinically documented arrhythmia was ventricular tachycardia arising from the LVOT area whereas AVNRT was the first tachycardia induced by programmed stimulation in the electrophysiologic laboratory, although this had not been documented clinically. Slow pathway ablation was done because of patient request and report of future recurrence of AVNRT in such patients [7]. Then LVOT-tachycardia was induced and ablated successfully because catheter ablation of one arrhythmia substrate did not prevent inducibility or clinical recurrence of the other [7].

Co existence of AVNRT and LVOT-tachycardia may be a no more than chance association. This suggestion appears to be supported by presence of different mechanisms for both each type of tachycardia. On the other hand, some debate was made for this hypothesis in Kautzner study explaining this combination by presence of common trigger in patients with combination of AVNRT and RVOT-tachycardia [7].

## Conclusion

Our case demonstrated: 1) Presence of rare coexistence of AVNRT and LVOT-tachycardia 2) Feasibility of successful ablation of combination of AVNRT and LVOT-tachycardia in the same patient during one session.
